# Phylogeography and Molecular Evolution of *Potato virus Y*

**DOI:** 10.1371/journal.pone.0037853

**Published:** 2012-05-24

**Authors:** José M. Cuevas, Agnès Delaunay, Johan C. Visser, Dirk U. Bellstedt, Emmanuel Jacquot, Santiago F. Elena

**Affiliations:** 1 Instituto de Biología Molecular y Celular de Plantas, Consejo Superior de Investigaciones Científicas-Universidad Politécnica de Valencia, València, Spain; 2 INRA-Agrocampus Ouest-Université Rennes 1, UMR1099 BiO3P (Biology of Organisms and Populations Applied to Plant Protection), Le Rheu, France; 3 Department of Biochemistry, Stellenbosch University, Matieland, South Africa; 4 The Santa Fe Institute, Santa Fe, New Mexico, United States of America; Institute of Infectious Disease and Molecular Medicine, South Africa

## Abstract

*Potato virus Y* (PVY) is an important plant pathogen, whose host range includes economically important crops such as potato, tobacco, tomato, and pepper. PVY presents three main strains (PVY^O^, PVY^N^ and PVY^C^) and several recombinant forms. PVY has a worldwide distribution, yet the mechanisms that promote and maintain its population structure and genetic diversity are still unclear. In this study, we used a pool of 77 complete PVY genomes from isolates collected worldwide. After removing the effect of recombination in our data set, we used Bayesian techniques to study the influence of geography and host species in both PVY population structure and dynamics. We have also performed selection and covariation analyses to identify evolutionarily relevant amino acid residues. Our results show that both geographic and host-driven adaptations explain PVY diversification. Furthermore, purifying selection is the main force driving PVY evolution, although some indications of positive selection accounted for the diversification of the different strains. Interestingly, the analysis of P3N-PIPO, a recently described gene in potyviruses, seems to show a variable length among the isolates analyzed, and this variability is explained, in part, by host-driven adaptation.

## Introduction

RNA viruses are characterized by large population sizes, fast replication and high mutation rates, which results on a huge evolutionary potential [1]. In addition, two types of genetic exchange, *i.e.*, reassortment in multipartite viruses, and recombination in either segmented or unsegmented viruses, may significantly contribute to speed up adaptation [2]. Not surprisingly, RNA viruses appear to be the most frequently associated pathogens to new emergent diseases, and this potential has become a major concern in agriculture, where some recent examples of catastrophic emergence have been described [3], [4].

PVY consists of a single-stranded positive sense RNA genome of about 10000 bases in length [5]. The genome has terminal untranslated (UTR) regions flanking a single large open reading frame. The viral polyprotein is cleaved by three virus-encoded proteases (P1, HC-Pro and NIa-Pro) into ten products (P1, HC-Pro, P3, 6K1, CI, 6K2, VPg, NIa-Pro, NIb, and CP) [6]. An additional peptide, P3N-PIPO, is translated from an overlapping ORF after +2 frameshifting of the P3 cistron [6]. PVY is type-member of the genus *Potyvirus* in the family *Potyviridae* [7] and is probably the most widespread virus infecting potatoes, as well as other crops, such as tobacco, tomato and pepper. It was originally classified into strain groups (*e.g*., PVY^C^, PVY^O^ and PVY^N^) according to biological properties or genome sequences [8], [9]. Later on, recombinant variants (*e.g*., PVY^NTN^ and PVY^N^-W) within the PVY^N^ group were identified [10]. More recently, additional PVY genomic organizations coming from previous recombinant forms have been described [11], [12]. Recombination is common in potyviruses [13] and is likely to impact the evolution of PVY populations, since most of the currently circulating isolates are recombinant forms.

For this study, the complete coding sequences of 68 worldwide PVY isolates, for which collection date, host and geographical origin were available, were retrieved from GenBank (Table S1). In addition, nine South African isolates collected during the period 2005–2010 were included in the analysis. We sought to shed some light on the epidemiological and evolutionary dynamics of PVY at a worldwide level. First, we evaluated the impact of recombination in our samples in order to remove its effect in subsequent analyses. Then, we performed Bayesian Markov chain Monte Carlo (MCMC) coalescent analyses to study the effect of local adaptation and host species in the observed diversity. Finally, we applied selection analyses to elucidate its influence on PVY evolution.

## Results and Discussion

### Removing the effect of recombination from PVY isolates dataset

**Figure 1:**
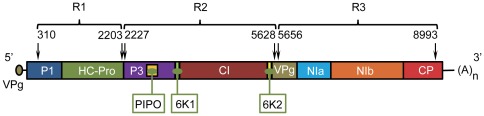
Schematic representation of the PVY genome, including UTR regions and gene distribution. The three regions (R1, R2 and R3) defined to perform the present study are indicated above and numbered ignoring the 5′ UTR.

Recombination has been commonly described in PVY genomes, where some genomes of recently described isolates are mosaics from previously described PVY genomes [11], [12], [14], [15]. As expected, recombination events detected by at least three of the eight statistical methods implemented in RDP3 [16] corroborated these previous results [11], [12], [14], [17], [18] (Table S2). Besides, a new additional recombination event was detected in the genome of isolate N Nysa encompassing the region 6099–8896 (with isolates GG517_128 and NN300_41 as the major and minor parents, respectively). No significant evidence for recombination was found in 19 out of the 77 isolates. By contrast, 17 isolates showed significant evidences for recombination breakpoints different from the four more commonly described ones [12] at positions 310, 2215, 5642, and 8996 (numbering excluding the 5′-UTR, as explained in Material and Methods) and therefore these isolates were removed from subsequent analyses. For the remaining data set, we divided the whole alignment into three recombination-free regions, named as R1, R2 and R3, ranging from 310 to 2202 (most P1 and HC-Pro), from 2227 to 5628 (almost the entire P3 to the 5′ part of VPg), and from 5656 to 8991 (most VPg to most CP) positions, respectively (Figure 1). Subsequent analyses were performed independently for these three genomic regions.

### Lack of a time stamp

Bayesian coalescent estimates were performed in parallel for the three defined PVY genomic regions. In our data set, temporal structure was not present for any of the three regions (see Material and Methods), precluding us to estimate evolutionary rates and divergence times. In this sense, it is usual that PVY field isolates are yearly propagated and maintained in potato or tobacco plants in greenhouses [17], [19]–[21]. Besides, it is reasonable to suppose that at least some of the sequences deposited in GenBank come from isolates expanded into experimental hosts and greenhouse conditions before sequencing, this fact being potentially more important for older isolates [19]. Consequently, it is logic to conclude that isolate collection dates can be misleading, thus complicating the potential detection of a temporal signature.

### Phylogeography analyses

**Figure 2:**
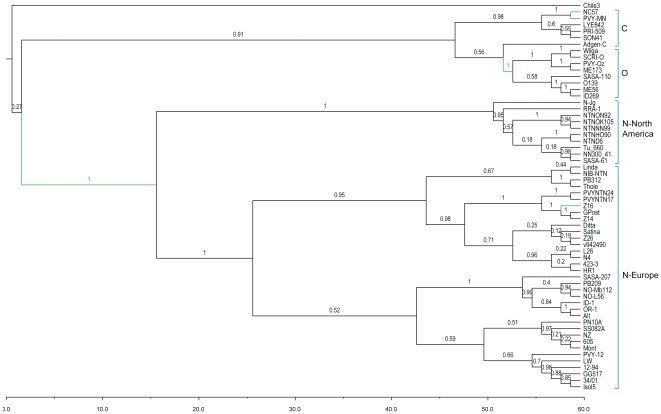
MCC phylogeny of 60 PVY isolates for the R1 region. The tree was calculated from the posterior distribution of trees generated by Bayesian MCMC coalescent analyses with BEAST [55]. Posterior probabilities are indicated above branches. Branches detected to be under positive selection are shown in green.

Again, these analyses were performed in parallel for R1, R2, and R3 regions. Figure 2 shows the MCC tree obtained for R1 region. For comparison, MCC trees for R2 and R3 regions are provided in Figures S1 and S2, respectively. For R3 region, isolates 12–94 and 34/01 are identical in sequence, and only the second one is shown in the tree (Figure S2). A previous phylogenetic study dealing with a similar pool of worldwide isolates, with the exception of the South African isolates incorporated here, used a similar strategy [14]. They defined three regions for the analyses considering the same recombination breakpoints we have used here, but with smaller sizes, and performed maximum-likelihood reconstructions. Our results were very similar in the sense that the three main strain groups (PVY^N^, PVY^O^ and PVY^C^) became clearly defined, and that PVY^N^ strain split into two well-supported sublineages (named as N-Europe and N-North America). Besides, the observed differences at tree topology among the three regions suggest that many European and North American isolates were recombinant (Figures 2, S1 and S2). Also, star phylogenies were commonly observed in the PVY^N^ and PVY^O^ lineages, indicating a recent origin with minimal selection [22]–[24]. Regarding South African isolates, we here corroborated previous results obtained analyzing only the CP gene [25]. South African isolates fell into both PVY^N^ sublineages and PVY^O^ lineage, besides showing recombinant PVY^N^-PVY^O^ forms. With respect to the PVY^C^ strain, the two tobacco isolates (PVY-MN and NC57) formed a well-supported clade, whereas the location of the other four isolates was less well defined. In particular, the potato isolate Adgen-C clearly fell into the C clade for R2 and R3 regions, but was located at a basal position between PVY^C^ and PVY^O^ clades for region R1. Besides, phylogenetic relationships for the remaining three isolates only were well defined for the R3 region, where the tomato isolate LYE84.2 formed a cluster with tobacco isolates, whereas PRI-509 and SON41 (isolated from potato and black nightshade, respectively) also formed a well-supported clade. Finally, as recently described, isolate Chile3 is at the base of the phylogenetic tree (Figure 2), thus becoming the most ancestral isolate, besides being the only non-potato isolate falling outside the PVY^C^ strain clade [26].

Taking into account that phylogenetic MCC trees do not suggest a clear effect of geographic structure in the genetic diversity of PVY at the continental level, we decided to test for this in a more quantitative manner. For commercial and geographical reasons, isolates from Canary Islands and Syria were included into the European group. For the same reasons, isolates from New Zealand and Chile were not included into any continental group. We calculated three summary statistics (association index *AI*, parsimony score *PS* and maximum monophyletic clade size *MC*) to describe the correlation between the geographic and the phylogenetic relationships from the posterior distribution of genealogies generated by the Bayesian coalescence analyses. Significant signatures for geographic structure in the diversity of PVY genomes were observed for the three regions analyzed when grouped by geographic origins (Table 1), as shown by the significant *AI* and *PS* values. All continental groups with the exception of the European one, showed differentiated subpopulations (significant *MC* values) and no inference was possible for some single isolates unassigned at a continental level due to their insufficient sample size. Japanese and South African isolates consisted of clearly differentiated subpopulations for the three regions analyzed. However, North American isolates only showed significant *MC* values for R1 and R2 regions. This difference in population structure for each genomic region was well accounted for by the effect of recombination, as mentioned above, since many North American and European isolates were recombinant between regions R2 and R3. Consequently, recombination seems to be masking the geographical origin of PVY isolates. In fact, due to this promiscuity, it has been proposed that the country (or location) of newly described isolates be considered as secondary, since global trade is promoting an increasing worldwide dispersion of PVY isolates [9].

**Table 1:** Analysis of the geographic and host effect on the population structure of PVY isolates for the three genomic regions under study.

Analyses	Association statistics	Test value			*P*		
		R1	R2	R3	R1	R2	R3
Geographic regions	*PS*	29.9725	30.2605	29.6408	<0.001	<0.001	<0.001
	*AI*	4.7758	4.6144	4.7875	<0.001	<0.001	<0.001
Europe	*MC*	2.3042	2.2272	2.2736	0.1700	0.1700	0.25
Japan	*MC*	1.0992	1.0988	1.0949	0.0099	0.0099	0.0099
North America	*MC*	2.5342	2.5425	2.7597	0.0099	0.0099	0.3999
South Africa	*MC*	1.3259	1.2988	1.3360	0.0099	0.0099	0.0099
Chile	*MC*	NA^1^	NA^1^	NA^1^			
New Zealand	*MC*	NA^1^	NA^1^	NA^1^			
Host species	*PS*	4.9775	4.9845	4.9999	0.0099	<0.001	<0.001
	*AI*	1.0503	0.9879	1.0682	<0.001	<0.001	<0.001
Potato	*MC*	13.0111	12.8143	13.0830	0.0099	0.0199	0.0099
Tobacco	*MC*	1.0217	1.0154	1.0000	0.0199	0.0099	0.0099
Tomato	*MC*	NA^1^	NA^1^	NA^1^			
Pepper	*MC*	NA^1^	NA^1^	NA^1^			
Black nightshade igrum	*MC*	NA^1^	NA^1^	NA^1^			

1 insufficient sample size (*n*<2).

To check the potential effect of host-driven adaptation, we computed the above summary statistics again but now using host as grouping variable. A significant effect of host in the distribution of PVY variability was also observed for the three regions under study (Table 1). In this case, the differentiation was due to two subpopulations of isolates derived from potato and tobacco, whereas no inference was possible for single isolates from tomato, pepper and black nightshade due to the low number of sequences from these hosts. However, we interpret these results with caution, because most isolates in our data set are from potato, and then the significance of *AI* and *PS* values could be a consequence of the global distribution of the same state across most of the branches in the tree [27]. It can de deduced however, that the phylogenetic structure was better explained by differences in hosts than by geographic distance. For example, four out of the five non-potato isolates included in our data set fall into the same clade, which corresponds with the PVY^C^ strain (Figure 2), a clustering that is not compatible with a common geographic origin since these isolates have quite diverse origins from Europe and North America. Finally, as mentioned before, temporal structure was not detected in our data set, maybe due to the greenhouse maintenance routines of some isolates before sequencing. Consequently, our results suggest that the phylogenetic signal associated with geographic and host structure is stronger than that relative to temporal structure.

Additionally, we wanted to examine whether potential differences in genetic variability could account for differences in population structure. To do so, we obtained nucleotide diversity estimates for each subpopulation showing significant *MC* values and compared them with global estimates (unpaired *t*-tests, results not shown). Regarding the influence of geographic structure, nucleotide diversity estimates for the different subpopulations were significantly lower than those from the global data set in most of the cases (Table S3). However, from a qualitative point of view, these estimates were quite similar, except for the Japanese subpopulation, which differed more than one order of magnitude for the three regions analyzed. Consequently, the apparently clear differentiation of the Japanese subpopulation could be explained as a typical example of founder effect in an insular environment [28]. With respect to the influence of host structure (Table S3), despite existing significant differences between subpopulation and global nucleotide diversity estimates, these differences were even smaller than for geographic structure in some cases, which supports the reliability of population structure analyses at host level.

### Detecting amino acid sites under different selective pressures

After evaluating the influence of geographic origin and host in the evolutionary diversification of PVY, we wished to assess the strength of selective forces behind these two factors. To do so, we estimated the differences in nonsynonymous and synonymous substitutions rates per site, *d_N_−d_S_*, using two complementary methods, FEL and IFEL [29], [30]. Table 2 shows the distribution of codon positions under purifying, neutral and positive selection for the different genes (partial or complete) included in our data set. The number of codons (when classified as selected or neutral) differs among ORFs (homogeneity test for FEL data: χ^2^ = 67.161, 9 df, *P*<0.001; homogeneity test for IFEL data: χ^2^ = 54.176, 9 df, *P*<0.001), indicating the existence of different selective pressures among genes [8]. While P1, P3, 6K1, 6K2, and CP genes have more neutral codons than expected from the marginal distributions, the remaining genes show more codons under selection than expected. Purifying selection is the main evolutionary force [31], although the distribution of positions do not show any clear concentration along the ORFs, which suggests that there are no domains that are particularly constrained. Only 11 positions were detected to be under positive selection (Table 2), three positions of which were located in a small region of CP (2922, 2981 and 2987), two in P1 (138 and 247) and P3 (900 and 919) and four in 6K1, CI, VPg, and NIb, respectively. Taken together, the reduced number of positively selected sites precludes us from drawing a conclusion regarding the potential existence of specific domains involved in adaptation. Consequently, the evolutionary dynamics of PVY seems to be strongly associated to purifying selection in combination with neutral evolution and some sporadic events of positive selection.

**Table 2:** Results of the codon selection analyses.

			FEL			IFEL			
Gene	Region	Sites	Negative	Neutral	Positive	Negative	Neutral	Positive	Location
P1*	104–275	172	54	116	2	41	129	2	138**, 247**
Hc-Pro*	276–734	459	188	271		113	346		
P3*	743–1105	363	120	242	2	66	296	2	900**, 919**
6K1	1106–1157	52	20	32		7	44	1	1150
CI	1158–1791	634	282	352		148	485	1	1404
6K2	1792–1843	52	20	32		9	43		
VPg*	1844–1876, 1886–2031	179	62	116	1	40	138	1	1966**
NIa-Pro	2032–2275	244	106	138		60	184		
NIb	2276–2794	519	193	326		110	408	1	2508
CP*	2795–2997	203	39	163	1***	19	182	2	2922, 2981, 2987

For each gene, the total number of codons, as well as those detected to be under negative, neutral or positive selection with both FEL and IFEL methods are given. The last column indicates the location of positively selected sites.

* partial genes;

** sites detected to be under positive selection by both FEL and IFEL methods;

*** FEL method detected position 2981, whereas the other two positively selected positions at the CP gene were detected with IFEL method.

In order to shed more light on the selective forces acting on PVY, we also assessed coevolution events among different amino acid sites [32]. Covariation was detected in 42 positions along six cistrons (Table 3), with the P3 gene showing the highest number of covarying positions (15). Interestingly, ten out of the 14 positions in this gene fall into the sequence coding for the P3N-PIPO protein [6], and this overlap could account for the sequence constraints detected. P3N-PIPO is embedded into the P3 cistron, translated in the +2 reading frame, and encodes a predicted protein of unclear length in PVY, since it has been described to have two lengths, 75 [6] and 76 (erroneously indicated in that study as residue 77) amino acids [19]. This variability in length will be discussed in the next section.

**Table 3:** Results of covariation analysis.

ORF	Codon position											
P1	111	121										
	214	241										
HC-Pro	286	289	305	310	369	378						
	321	379	395	569	622	630						
P3	761	1094										
	901	**916**	**919**	**936**	**939**	**943**	**946**	**947**	**972**	**974**	**983**	1034
CI	1172	1214	1323	1404	1428	1431	1631	1639				
NIa-Pro	2058	2155										
NIb	2295	2373										

For P1, HC-Pro and P3 ORFs, two groups of covariation were detected (indicated in different rows). Positions falling into the PIPO region codifying for the P3N-PIPO cistron are indicated in bold.

In order to discern the biological relevance of positively selected and covarying positions in terms of host-driven adaptation, we analyzed their potential differences in amino acid composition between potato and non-potato isolates (Tables S4 and S5), although the small number of non-potato isolates precluded us from drawing strong conclusions. Regarding positively selected sites (Table S4), the same predominant amino acid was observed in five positions (919, 1150, 1404, 1966, and 2922) for potato and non-potato isolates. For the remaining positions, the predominant residue for non-potato isolates was always present at low frequency in potato isolates, except for residue 2987, showing a certain level of variability in this position in both groups. When looking at the covarying positions (Table S5), clear differences in the predominant amino acid for potato and non-potato isolates were mainly observed for P1 and HC-Pro genes, whereas the other genes showed more frequently the same predominant residue in both groups. Globally, specific residues of non-potato isolates were usually present at low frequencies, whereas those specific of potato isolates were commonly present at high frequencies (Tables S4 and S5). Consequently, particular residues accounting for host adaptation were not observed in any case, since most of the positively selected and covarying positions shared common residues at variable frequencies.

Finally, variable selection analyses per branches could help us to establish an association between selective events and the obtained phylogenies. First of all, it is worth mentioning that the CODEML program was only used to obtain the most likely evolutionary model and the parameters associated. Consequently, those positions detected to be under positive selection using CODEML were ignored and not included in this study, since it has been previously shown that the statistical test for positive selection implemented in this program is far less conservative than those used by the FEL and IFEL methods [33]. Results from branch selection analyses are summarized in Table 4, which shows the positive selection events at internal and terminal branches for the three regions under study. Branches under positive selection are also indicated in Figures 2, S1 and S2. Several significantly covarying sites (shown in Table 3) are included in the detected subregions (Table 4), whereas none of the positively selected codons (Table 2) are included in these subregions. Regarding the genome distribution of the 12 positively selected branches (ten terminal and two internal branches), four fell into the P1 protein, three into the P3 protein, and one into the HC-Pro, CI, NIa-Pro, NIb, and CP proteins, respectively, which is congruent with the previous codon selection and covariation analyses (Tables 2 and 3). Selection at terminal branches only provides information about particular events in single isolates and it is worth noting that most of these branches belong to non-potato isolates. Isolate NC57 is the one showing the strongest effect of selection, since its branch was positively selected in the analyses of the three regions under study, showing five involved subregions (located in the P1, P3 and NIb proteins) along its genome (Table 4). To a lesser extent, the branch leading to PVY-MN isolate was positively selected in three subregions belonging to P1 and CP proteins, the branch leading to Z16 isolate in two subregions from HC-Pro and P3 proteins, whereas the branch leading to Chile3 isolate showed positive selection in two subregions from P3 protein. The other two isolates (GPost and SON41) showed positive selection in single subregions from CI and NIa-Pro proteins, respectively. It is worth noting that a subregion from isolate NC57 included five positions belonging to a previously detected covariation group in the P3 protein (Tables 3 and 4), all these positions falling into the embedded P3N-PIPO cistron. Consequently, the agreement between covariation and branch selection analyses corroborated the potential role of P3N-PIPO as a target of selection. In addition, several selected subregions from terminal branches also included covarying positions at P1, P3 and NIb proteins (Table 4), supporting its potential selective role.

**Table 4:** Results of selection analyses per branches.

Region	Branch	Subregion	ORF	Covariation
R1	PVY^N^ strain	116–124	P1	121
		160–165		
		205–214		214
		240–245		241
	PVY^O^ strain	116–124		121
		208–214		214
	PVY-MN	151–157		
		209–214		214
	NC57	199–209		
		213–223		214
		264–269		
	Z16	585–591	HC-Pro	
R2	Z16	757–763	P3	761
	NC57	931–949		936, 939, 943, 946, 947
	Chile3	1053–1060		
		1094–1100		1094
	Gpost	1436–1442	CI	
R3	SON41	2247–2250	NIa-Pro	
	NC57	2370–2378	NIb	2373
	PVY-MN	2859–2865	CP	

For each region under study, branches showing positive selection, location of amino acid subregions involved and ORFs are indicated. For terminal branches, the name of the corresponding isolate is shown, whereas for internal branches the strain subjected to the selection event is indicated. Covarying positions detected in the previous analyses falling into the subregions providing a positive selection signature are shown in the last column. (*P*<0.0001 for all subregions).

Regarding isolate NC57, it is worth noting that a visual inspection allowed us to detect that four out of the five subregions showing selection signatures (subregions 199–209, 213–223, 931–949, and 2369–2379) match relatively well with locations in its genome where single-nucleotide indels (insertion or deletion, depending of the given region) are followed by additional compensatory single-nucleotide indels a few codons downstream that restore the standard reading frame of the polyprotein, while still creating a clearly different amino acid sequence in these regions. It is not possible to know if this is consequence of sequencing errors or adaptive events, but two plausible biological explanations may support the latter. Firstly, subregions 199–209 and 213–223 are partially overlapping with other subregions for which selective signatures have been detected in the terminal branch leading to PVY-MN isolate of strain PVY^C^ and in the two internal branches leading to PVY^N^ and PVY^O^ strains (Table 4), providing evidence of the global selective pressure affecting this P1 region. Secondly, covarying site 214, included in the potentially biased subregion 213–223, is also included in subregions from other branches, supporting its biological relevance. However, it may be argued that covariations in subregion 931–949 have been exclusively detected for the branch leading to isolate NC57 and that they may just be local sequencing errors. This argument can be easily discounted because (i) covariation analyses consider the pattern of the global phylogeny and not individual sequences when detecting covariation groups (Mario A. Fares, pers. commun.) and (ii) seven positions in the same covarying group fall outside this subregion (three upstream and four downstream; Table 3), and thus would hardly be affected by the claimed sequencing errors. Subregion 2369–2379 was also only detected in the branch leading to isolate NC57. This subregion included covarying site 2373 but not site 2295 (Table 3), and, again, potential sequencing errors during the characterization of isolate NC57 could not account for the detection of this covariation event. In summary, we cannot fully discern from our results if amino acid changes at these two NC57 subregions are artifacts or due to adaptive events. However, all the above information supports the credibility of the adaptive explanation.

At this point, we sought to analyze the biological consequences of observed positive selection at the two internal branches (Figure 2), whose involved subregions fell into the P1 protein (Table 4). Both branches showed a coincident subregion (positions 116–124) and a partially overlapping subregion, whereas two other subregions were detected at the branch leading to the PVY^N^ clade (Figure 2). These different subregions supporting positive selection in the two observed internal branches included three covarying positions (121, 214 and 241), and it was particularly remarkable in the case of position 214, since it also was included in subregions from PVY^C^ strain isolates PVY-MN and NC57, which stressed its potential adaptive role in the three PVY strains. In conclusion, these common subregions between the two internal branches, together with the other two close but non-overlapping subregions in the P1 protein, provided clear phylogenetic evidence of the initial differentiation of the clade including PVY^N^ strain isolates, and the subsequent separation between PVY^C^ and PVY^O^ strains (Figure 2).

To establish the relationship between internal branch selection and molecular changes associated, we obtained the amino acid composition of involved subregions for the three main clades (*i.e.*, PVY^N^, PVY^O^ and PVY^C^), regardless the internal branch to which they belonged (Table S6). By doing so, we could observe the potential discriminative power of each position for the global phylogeny and not only between the clade indicated by the selected branch and the rest of the phylogeny. Isolate Chile3 was not included in this analysis, since it was not assigned to any phylogenetic group (Figure 2). First of all, it is worth mentioning that amino acid residues were much more conserved for the group of isolates from PVY^O^ strain, much more diverse for isolates from PVY^C^ strain, and showing an intermediate level of variability in the PVY^N^ strain (Table S6). As expected, these differences in variability clearly correlated with the number of specific residues from each strain. For each given amino acid position, the exclusive residues from PVY^N^ and PVY^O^ strains were usually at high frequencies or even conserved, whereas those from PVY^C^ strain were usually at low frequencies. Taken together, the high level of polymorphism and the often low frequency of specific residues from PVY^C^ strain could be due to differential selective pressures, since the six isolates included in this strain were collected from four different hosts. Different categories of positions could be assigned depending on their discriminatory ability among strains. Whereas six positions (116, 121, 162, 206, 240, and 243) discriminated very well between the PVY^N^ strain and the remaining two strains, positions 122, 209 and 211 were fully discriminant between PVY^O^-PVY^C^, PVY^O^ and the rest and PVY^C^ and the rest, respectively. Interestingly, positions 160 and 210 were fully discriminant among the three strains and four other positions (120, 214, 241, and 242) were almost fully discriminant. Similarly with previous analyses seeking to detect discriminant positions between potato and non-potato isolates, we observed that position 116 was the only candidate. In fact, when analyzing position 116 in detail, we observed that the only amino acid that was shared by PVY^O^ and PVY^C^ strains belongs to isolates PRI-509 and Adgen-C, which are the only potato isolates of the PVY^C^ strain. Consequently, position 116 seems to be highly discriminant between potato and non-potato isolates. However, it is important to stress that this conclusion was obtained whilst disregarding the influence of the non-potato isolate Chile3 (not included in this analysis, as mentioned above), which shares the same amino acid with PRI-509 and Adgen-C (and isolates from the PVY^O^ strain, indeed) at position 116. The unique biological and genomic properties of isolate Chile3 suggest its earliest divergence during PVY evolution, besides that it belongs to a new strain [26] and therefore, in view of the basal position of isolate Chile3 in the phylogeny, this may suggest that this amino acid (S) is rather the plesiomorphic state in this position, than a host specific adaptation.

### Analyzing biological consequences

Furthermore, we tried to associate our selection and covariation analyses with regions or functions of biological relevance in PVY. Intra-molecular covariation can take place in order to maintain RNA or protein secondary structures, besides physical interactions with other viral and non-viral proteins. In this sense, it has been described for different potyviruses that the P3 protein (alone or in conjunction with P3N-PIPO) interacts with the small and large subunits of RubisCO [34]. P3 and P3N-PIPO are also involved in intercellular movement of potyviruses in infected plants [35], [36]. Also, multiple virulence determinants have been described in P3, even overlapping with P3N-PIPO in a few cases, but this virulence has always been associated with P3 [37], perhaps simply because P3N-PIPO was not known before. In addition, it has been shown that CP and HC-Pro proteins from PVY, CI protein from *Plum pox virus* and P1 protein from *Soybean mosaic virus,* also interact with different chloroplast proteins [38]–[41]. In the particular case of P1 protein, the positively selected (at branch and codon level) and covarying sites (Tables 2, 3 and 4) detected here are relatively close each other and outside the region potentially involved in this interaction [41], although an indirect effect in terms of protein structure stabilization cannot be discounted. Besides, the main function of P1 protein is as a protease. This activity is located within the 147 C-terminal residues (codons 129–275 in the P1 cistron) [42], and hence positively selected positions 138 and 247, covarying positions 214 and 241, and several subregions detected in branch selection analyses, fall into this domain, thus strongly suggesting a potential role of positive selection in optimizing the protease activity upon, *e.g.* host changes. The positively selected position 1966 (codon 123 in the VPg cistron) is located in the central part of the VPg protein, which is known to determine virulence to a series of alleles of resistance in pepper that code for variants of the eukaryotic translation initiation factor 4E (eIF4E) [43]–[45]. Taking into account that several functional molecular models involve the eIF4E-VPg physical interaction to determine resistance or virulence properties in plant-virus interactions [46], [47], the potential effect of the positively selected site described here deserves further investigation. Regarding the CP protein, a recent study seeking for positive selection has observed a significant relationship between two positively selected positions (2819 and 2862) and their adaptive advantage in terms of virus accumulation and infectivity [48]. In our study, we have observed three positively selected sites located downstream of these previously reported positions, but the joint results of both studies appear to point to the potential adaptive relevance of this region. In this regard, CP is involved in vector transmission [49] and in systemic plant colonization [50], [51], thereby becoming a potential target of selection at both vector and plant levels. However, the methods employed for detecting positive selection were not exactly the same in both studies and the sequence data sets had very limited overlap, thereby possibly accounting for the different results.

The combination of results obtained at codon, branch selection and covariation analyses led us to conclude that selective forces are not driving PVY evolution in terms of host specific adaptation but mainly promoting the emergence and spread of the current strains. Only positions 160 and 210 explained PVY phylogeny at the strain level, whereas position 116 partially explained the phylogeny in terms of host adaptation dynamics. The main determinants of PVY differentiation seem to be located in the P1 protein, the least conserved potyviral protein, and postulated as likely involved in the adaptation of potyviruses to a wide range of host species [52].

### Potential length variability in P3N-PIPO

P3N-PIPO was recently described as a protein of variable length among potyviruses [6]. After detailed inspection of P3N-PIPO sequences, we found that they varied in length among PVY isolates, *i.e.* at the intra-specific level (Figure S3). It has been postulated recently that P3N-PIPO encodes a protein of 76 amino acids in PVY [19] (erroneously indicated in that study as residue 77) and the stop codon (UAA) leading to this size is present in all our isolates. However, we have found additional stop codons that could be involved in promoting differences in P3N-PIPO length. Forty isolates (out of the 60) also present a stop codon (UAA) at position 76 (Figure S3). Seventeen isolates present another stop codon (UGA) at position 73; and one of these isolates (PVY-Oz) presents a second stop codon at position 76. Isolates Adgen-C and SASA-207 show this stop codon at position 76 plus an additional one at positions 74 and 62, respectively. Finally, the stop codon at position 77 is the only one present in isolates N4, LYE84.2, Chile3, and PRI-509. Two out of these four isolates belong to the PVY^C^ strain, which is particularly interesting, because our data set only includes six isolates from this strain (Figure 2). In fact, if we construct a two-by-two contingency table by assigning isolates according to whether they have only the stop codon at position 77 or additional ones and whether they belong to the PVY^C^ strain or to any other, a significant excess of PVY^C^ isolates having only one stop codon exists (Fisher's exact test, *P* = 0.046). Besides this, isolate Chile3 has been postulated as belonging to a new lineage [26], although it is also included within the PVY^C^ strain in accordance with its peculiar host range [18], which would even give more support to our results. Consequently, these differences in size could be partially accounted for by phylogenetic relationships. However, additional analyses of sequence databases from other potyviruses, besides complementary functional studies are necessary to discern the relevance of this potential intra-specific plasticity in P3N-PIPO length.

### Concluding remarks

In summary, the analyses presented here have shed some light on the spatial genetic structure driving PVY evolutionary dynamics at a worldwide level, besides giving an indication of the gene-specific selective forces involved in PVY evolution. However, greater numbers of isolates from different hosts will have to be included in future studies before definite conclusions can be made. The joint analyses of results such as those presented here, together with symptomatology and serology studies may well contribute to the future development of more effective and durable control strategies.

## Materials and Methods

### Alignment of the genomes and recombination analyses

UTRs were removed from the complete genome sequences. Nucleotide sequences were then translated and amino acid sequences were aligned with MUSCLE [53] as implemented in MEGA5 [54]. Short insertions of one or two codons were observed in some sequences and removed from the alignment. This alignment was used as guide to produce the corresponding codon alignments that were employed for subsequent analyses. Consequently, nucleotide positions mentioned in this study always disregarded the 5′ UTR and started at the first codon position of the PVY large ORF. In addition, codon positions were given as the corresponding amino acid positions in the polyprotein.

RDP3 version 3.42 [16] was used to detect putative recombination breakpoints with default configuration, apart from the option of linear sequence and of disentangling overlapping signals. Only those breakpoints detected by at least three out of the eight methods implemented in RDP3 were accepted.

### Testing evolutionary dynamics

In order to check the existence of a temporal structure in our data set, we used PATH-O-GEN version 1.3 (tree.bio.ed.ac.uk/software/pathogen). First, we estimated the model of nucleotide substitution that best fitted the data using the application JModelTest as implemented in the PHYLEMON 2.0 webserver (phylemon.bioinfo.cipf.es/index.html). For the three regions, the best model was GTR+*Γ*_4_+I, and trees constructed with this model were used as input in PATH-O-GEN to perform a linear regression between the genetic distance from the root and the collection date for each sequence. Temporal structure was not observed in our data set.

Data were analyzed using the GTR+*Γ*_4_+I model using the Bayesian MCMC approach implemented in BEAST version 1.6 [55]. Due to the absence of temporal structure, sampling times were not specified when constructing the input file for BEAST, and the mean rate of molecular clock model was fixed to one. We tested the four clock models implemented in BEAST (strict, uncorrelated exponential, relaxed uncorrelated lognormal, and random local clock models) for the three regions. Substitution rates were estimated using the relaxed uncorrelated exponential clock model for the R1 region, and the relaxed uncorrelated lognormal clock model for the R2 and R3 regions, since Bayes factor provided a strong support in comparison with the alternative models. The MCMC was run for 10^8^ generations to ensure convergence of all parameters. The inspection of posterior distributions and the estimation of the relevant evolutionary parameters were done with TRACER version 1.5 (tree.bio.ed.ac.uk/software/tracer). The first 10% of sampled trees were discarded as burn-in. Statistical significance of parameters was evaluated using the 95% credible interval, also known as highest probability density (HPD).

### Analyzing geographic structure and host origin

In order to estimate the maximum clade credibility (MCC) phylogeny, including its posterior probabilities, the posterior set of trees (showing branch lengths in substitutions) obtained in BEAST was used. To do so, TREEANNOTATOR version 1.6.1 (beast.bio.ed.ac.uk) was used with 10% of the trees discarded as burn-in. The reliability of the MCC tree was evaluated using 95% HPD confidence intervals. To elucidate the influence of the geographic origin and the host in PVY populations, we used BATS version 1.0b2 [27], which computes the parsimony score (*PS* [56]), the association index (*AI* [57]) and the maximum monophyletic clade size (*MC* [27]) statistics, besides assessing their significance. The first 10% of sampled trees were discarded as burn-in and 10^4^ randomizations were performed to estimate the null distributions of the three statistics. Finally, nucleotide diversity estimates were obtained for the whole data set and for significant cases in geographic origin and host analyses using DNASP [58]. By doing so, it is possible to check the potential influence of differences in nucleotide diversity in the detection of significant differences.

### Evaluating the strength of selection

Selective pressures at a codon level were estimated as the difference between nonsynonymous (*d_N_*) and synonymous (*d_S_*) substitutions rates per codon using the fixed-effects likelihood (FEL), and internal branches fixed-effects likelihood (IFEL) [29], [30]. These analyses were performed for the three regions defined in our study. To complement this codon specific analysis, we also performed additional analyses to detect selective constraints at a branch level for the phylogenies obtained at the three regions under study. To do so, first the CODEML program from PAML package 4.4 [59] was used to estimate the best codon substitution model and associated parameters. Two criteria were employed to assign the most likely evolutionary model: a likelihood ratio test (LRT), which compares the fit of two nested models to the data [60] and the Akaike information criterion (*AIC*), which allows to perform comparisons between non-nested models [61]. For each region, six models were compared: M0, M1, M2, M3, M7, and M8. For models M2, M3 and M8, the existence of positively selected codons is allowed as they incorporate a class of codons for which *ω* = *d_N_*/*d_S_* can take values >1. Parameters belonging to the most likely model, together with the codon frequencies estimated from the real data set, were employed to generate a set of 1000 random sequence alignments simulated by maximum likelihood under a known phylogenetic tree by using the EVOLVER program from PAML. This set of simulated alignments was finally used with SWAPSC program [62] to detect branch specific selection. This program includes an automatic windowing screening for selective constraints using the Kimura-based method of Li [63].

Additionally, intramolecular covariation analyses were carried out using CAPS version 1 [32]. To do so, the three regions comprising our study were fragmented into single complete or partial genes, and then translated. Subsequently, coevolution analyses were performed independently for each protein using default parameters for CAPS, but the threshold correlation coefficient between sites (*R*) and the number of random samples for the significance tests that were set up to 0.5 and 10^5^, respectively.

For those positions detected to be under positive selection or covariation, amino acid composition was obtained using the VESPA application [64] from the HIV sequence database (www.hiv.lanl.gov). The same was applied to subregions showing evidence of positive selection at internal branches.

## Supporting Information

Figure S1 **MCC phylogeny of 60 PVY isolates for the R2 region.** The tree was calculated from the posterior distribution of trees generated by Bayesian MCMC coalescent analyses with BEAST [55]. Posterior probabilities are indicated above branches. Branches detected to be under positive selection are shown in green.
(TIF)

Figure S2 **MCC phylogeny of 59 PVY isolates for the R3 region.** The tree was calculated from the posterior distribution of trees generated by Bayesian MCMC coalescent analyses with BEAST [55]. Posterior probabilities are indicated above branches. Branches detected to be under positive selection are shown in green.
(TIF)

Figure S3 **Amino acid alignment of P3N-PIPO in the isolates included in our data set.** Stop codons are highlighted in red.
(TIF)

Table S1 **PVY isolates used in the present study.**
(DOC)

Table S2 **Recombination events detected in PVY and bibliographic references.**
(DOC)

Table S3 **Genetic diversity estimates per geographic and host structure.**
(DOC)

Table S4 **Amino acid composition for potato (P) and non-potato (NP) isolates (55 and five, respectively) at positively selected codons.**
(DOC)

Table S5 **Amino acid composition for potato (P) and non-potato (NP) isolates (55 and five, respectively) at covarying codons.**
(DOC)

Table S6 **Amino acid composition for PVY^N^, PVY^O^ and PVY^C^ strain isolates (45, eight and six isolates, respectively) at the subregions showing evidence of positive selection for the branch leading to PVY^N^ isolates respect to the phylogeny of the R1 region.**
(DOC)
