# Supplementary figures and images for: Phylogeography and Molecular Evolution of Potato virus Y
Source: PLoS One. 2012 May 24;7(5):e37853. doi: 10.1371/journal.pone.0037853 (PMC3360008; doi:10.1371/journal.pone.0037853)

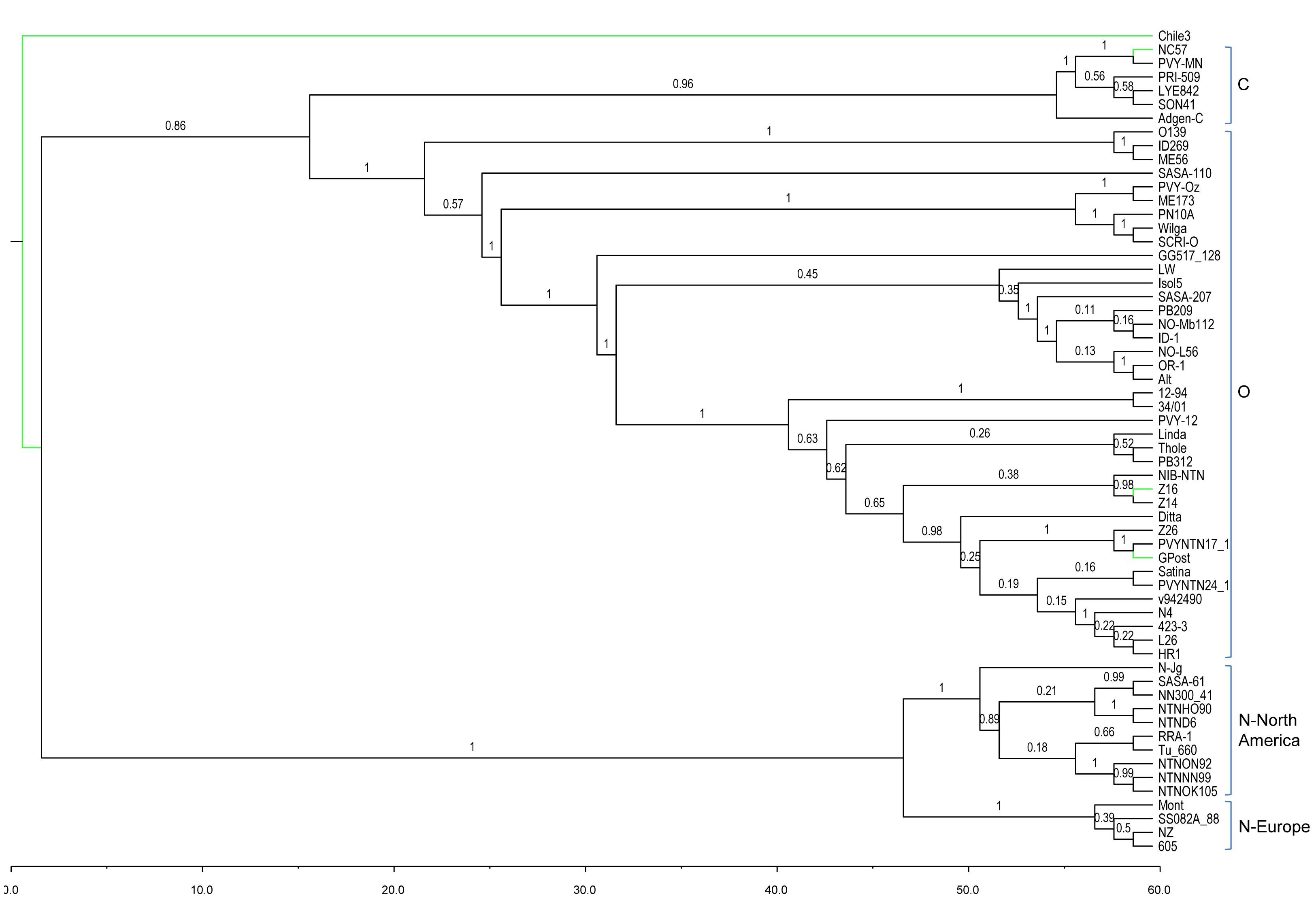

Supplement: Figure S1 — MCC phylogeny of 60 PVY isolates for the R2 region. The tree was calculated from the posterior distribution of trees generated by Bayesian MCMC coalescent analyses with BEAST [55]. Posterior probabilities are indicated above branches. Branches detected to be under positive selection are shown in green. (TIF) [file pone.0037853.s001.tif]

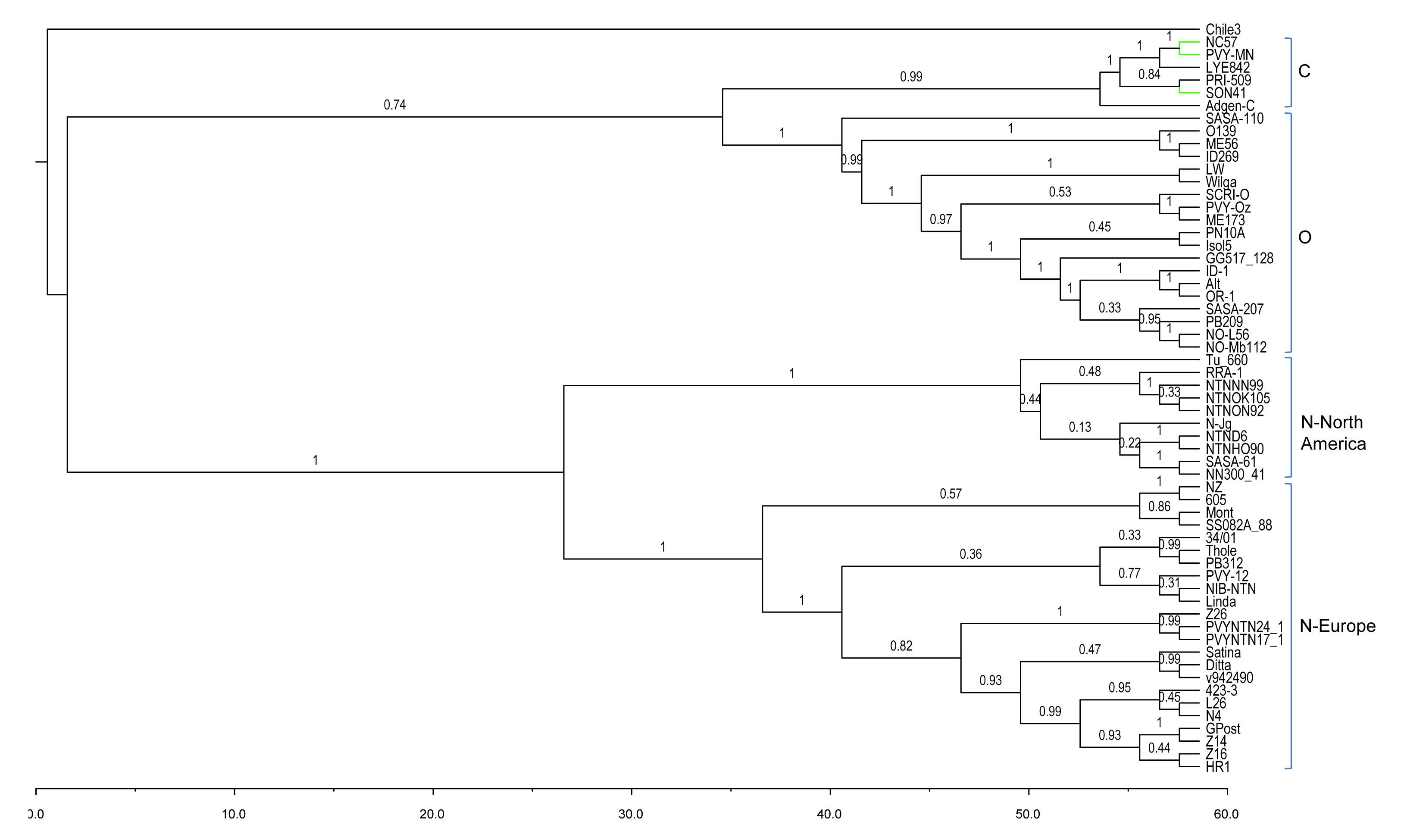

Supplement: Figure S2 — MCC phylogeny of 59 PVY isolates for the R3 region. The tree was calculated from the posterior distribution of trees generated by Bayesian MCMC coalescent analyses with BEAST [55]. Posterior probabilities are indicated above branches. Branches detected to be under positive selection are shown in green. (TIF) [file pone.0037853.s002.tif]

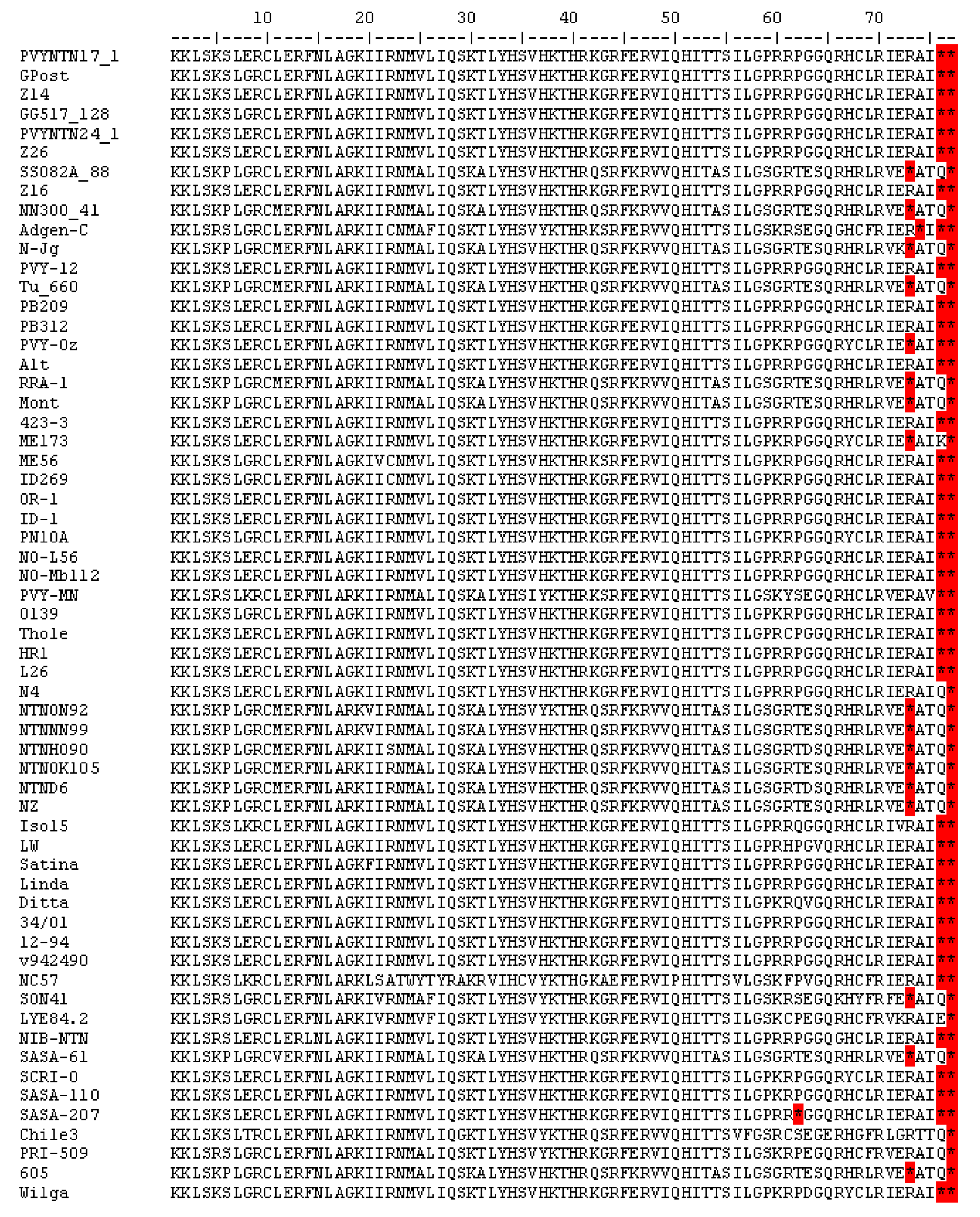

Supplement: Figure S3 — Amino acid alignment of P3N-PIPO in the isolates included in our data set. Stop codons are highlighted in red. (TIF) [file pone.0037853.s003.tif]
