# Supplementary material for: Phylogeography and Molecular Evolution of Potato virus Y
Source: PLoS One. 2012 May 24;7(5):e37853. doi: 10.1371/journal.pone.0037853 (PMC3360008; doi:10.1371/journal.pone.0037853)
Supplement: Table S1 — PVY isolates used in the present study. (DOC) [file pone.0037853.s004.doc]

| **Isolate** | **GenBank accession** | **Origin** | **Host** | **Collection date** |
| --- | --- | --- | --- | --- |
| PVYNTN17_1 | JN936429 | South Africa | *S. tuberosum* | 2007 |
| GPost | JN936420 | South Africa | *S. tuberosum* | 2010 |
| Z14 | JN936440 | South Africa | *S. tuberosum* | 2009 |
| GG517_128 | JN936419 | South Africa | *S. tuberosum* | 2005 |
| PVYNTN24_1 | JN936431 | South Africa | *S. tuberosum* | 2008 |
| Z26 | JN936442 | South Africa | *S. tuberosum* | 2005 |
| SS082A_88 | JN936433 | South Africa | *S. tuberosum* | 2005 |
| Z16 | JN936441 | South Africa | *S. tuberosum* | 2010 |
| NN300_41 | JN936422 | South Africa | *S. tuberosum* | 2005 |
| Adgen-C | AJ890348 | France | *S. tuberosum* | 2005 |
| N-Jg | AY166867 | Canada | *S. tuberosum* | 1991 |
| PVY-12 | AB185833 | Syria | *S. tuberosum* | 2003 |
| Tu 660 | AY166866 | Canada | *S. tuberosum* | 1991 |
| PB209 | EF026076 | USA | *S. tuberosum* | 2003 |
| PB312 | EF026075 | USA | *S. tuberosum* | 2003 |
| PVY-Oz | EF026074 | USA | *S. tuberosum* | 2003 |
| Alt | AY884985 | USA | *S. tuberosum* | 2002 |
| RRA-1 | AY884984 | USA | *S. tuberosum* | 2001 |
| Mont | AY884983 | USA | *S. tuberosum* | 2001 |
| 423-3 | AY884982 | USA | *S. tuberosum* | 2002 |
| ME173 | FJ643479 | USA | *S. tuberosum* | 2006 |
| ME56 | FJ643478 | USA | *S. tuberosum* | 2006 |
| ID269 | FJ643477 | USA | *S. tuberosum* | 2006 |
| OR-1 | DQ157179 | USA | *S. tuberosum* | 2001 |
| ID-1 | DQ157178 | USA | *S. tuberosum* | 2003 |
| PN10A | DQ008213 | USA | *S. tuberosum* | 2004 |
| NO-L56 | AY745492 | Canada | *S. tuberosum* | 1991 |
| NO-Mb112 | AY745491 | Canada | *S. tuberosum* | 1991 |
| PVY-MN | AF463399 | USA | *N. tabacum* | 2001 |
| O139 | U09509 | Canada | *S. tuberosum* | 1994 |
| Thole | M95491 | Hungary | *S. tuberosum* | 1993 |
| HR1 | FJ204166 | USA | *S. tuberosum* | 2007 |
| L26 | FJ204165 | USA | *S. tuberosum* | 2007 |
| N4 | FJ204164 | USA | *S. tuberosum* | 2007 |
| NTNON92 | AB331519 | Japan | *S. tuberosum* | 1997 |
| NTNNN99 | AB331518 | Japan | *S. tuberosum* | 1997 |
| NTNHO90 | AB331517 | Japan | *S. tuberosum* | 1997 |
| NTNOK105 | AB331516 | Japan | *S. tuberosum* | 1997 |
| NTND6 | AB331515 | Japan | *S. tuberosum* | 1997 |
| NZ | AM268435 | New Zealand | *S. tuberosum* | 2002 |
| Isol5 | AJ890350 | Germany | *S. tuberosum* | 2002 |
| LW | AJ890349 | Poland | *S. tuberosum* | 1970 |
| Satina | AJ890347 | Germany | *S. tuberosum* | 2002 |
| Linda | AJ890345 | Germany | *S. tuberosum* | 2004 |
| Ditta | AJ890344 | Austria | *S. tuberosum* | 1998 |
| 34/01 | AJ890342 | Poland | *S. tuberosum* | 2001 |
| 12-94 | AJ889866 | Poland | *S. tuberosum* | 1994 |
| v942490 | EF016294 | UK | *S. tuberosum* | 1994 |
| NC57 | DQ309028 | USA | *N. tabacum* | 1973 |
| SON41 | AJ439544 | France | *S. nigrum* | 1972 |
| LYE84.2 | AJ439545 | Canary Islands | *L. esculentum* | 1984 |
| NIB-NTN | AJ585342 | Slovenia | *S. tuberosum* | 1990 |
| SASA-61 | AJ585198 | UK | *S. tuberosum* | 1997 |
| SCRI-O | AJ585196 | UK | *S. tuberosum* | 1985 |
| SASA-110 | AJ585195 | UK | *S. tuberosum* | 1997 |
| SASA-207 | AJ584851 | UK | *S. tuberosum* | 1997 |
| Chile3 | FJ214726 | Chile | *C. baccatum* | 2005 |
| PRI-509 | EU563512 | The Netherlands | *S. tuberosum* | 1938 |
| 605 | X97895 | Switzerland | *S. tuberosum* | 1976 |
| Wilga | EF558545 | Poland | *S. tuberosum* | 1984 |
| SYR-NB-16 | AB270705* | Syria | *S. tuberosum* | 2006 |
| SYR-II-DrH | AB461453* | Syria | *S. tuberosum* | 2007 |
| SYR-II-Be1 | AB461452* | Syria | *S. tuberosum* | 2004 |
| SYR-II-2-8 | AB461451* | Syria | *S. tuberosum* | 2006 |
| HN2 | GQ200836* | China | *S. tuberosum* | 2007 |
| NE-11 | DQ157180* | USA | *S. tuberosum* | 2003 |
| Nnp | AF237963* | Italy | *C. baccatum* | 1992 |
| 261-4 | AM113988* | Germany | *S. tuberosum* | 2004 |
| Nicola | AJ890346* | Germany | *S. tuberosum* | 2001 |
| Gr99 | AJ890343* | Poland | *N. tabacum* | 1999 |
| 156var | AJ889868* | Germany | *S. tuberosum* | 2004 |
| 156 | AJ889867* | Germany | *S. tuberosum* | 2000 |
| SCRI-N | AJ585197* | UK | *S. tuberosum* | 1985 |
| N Nysa | FJ666337* | Poland | *S. tuberosum* | 1974 |
| SD1 | EU182576* | China | *N. tabacum* | 2007 |
| N-Egypt | AF522296* | Egypt | *S. tuberosum* | 2001 |
| Foggia | EU482153* | Italy | *L. esculentum* | 2007 |

*Isolates showing additional recombination breakpoints to those used to define the three regions under study, i.e., 310, 2215, 5642, and 8996 (excluding the 5’-UTR), and then removed for subsequent analyses. Underlined isolates in column one, newly described in this paper.
