# Supplementary material for: Phylogeography and Molecular Evolution of Potato virus Y
Source: PLoS One. 2012 May 24;7(5):e37853. doi: 10.1371/journal.pone.0037853 (PMC3360008; doi:10.1371/journal.pone.0037853)
Supplement: Table S2 — Recombination events detected in PVY and bibliographic references. (DOC) [file pone.0037853.s005.doc]

| Reference | Breakpoints | | | | | | | | | | | | | | | | | | | | | | |
| --- | --- | --- | --- | --- | --- | --- | --- | --- | --- | --- | --- | --- | --- | --- | --- | --- | --- | --- | --- | --- | --- | --- | --- |
| [8] | 495 |  |  |  |  |  |  | 2410 |  |  | 5807-5837 |  | 7880 |  |  | 8313 |  |  | 8709 | 8743 | 9165-9178 |  | 9450-9461 |
| [20] |  | 603 |  |  |  |  |  |  |  |  |  |  |  |  |  |  |  |  |  |  |  |  |  |
| [65] |  |  |  |  |  |  |  | 2412 |  |  | 5897 |  |  |  |  |  |  |  |  |  | 9170 |  |  |
| [12] | 500 | 600 | 645 |  |  |  | 2170 | 2390 |  |  | 5715 | 6720 | 7890 | 7940 | 8140 | 8320 | 8560 |  |  |  | 9170 | 9374 | 9460 |
| [15] |  |  |  |  |  | 2056-2632 |  |  |  |  |  |  |  |  |  |  |  |  |  |  |  |  |  |
| [14] |  |  |  |  |  |  |  |  |  | 3530 |  |  |  |  |  |  |  |  |  |  |  |  |  |
| [11] | 500 |  |  |  | 2000 | 2015-2696 |  | 2396-2412 |  |  |  |  |  |  |  |  | 8572 |  |  |  | 8853-9620 |  |  |
| [17] |  |  |  |  |  |  |  |  | 2521 |  | 5867 |  |  |  |  |  |  |  |  |  |  |  |  |
| [25] |  |  |  |  |  |  |  |  |  |  |  |  |  |  |  |  |  |  |  |  | 9171-9178 |  |  |
| [18] | 499 |  |  | 686 |  |  |  | 2414 |  |  | 5833 |  |  |  |  |  |  | 8604 |  |  |  |  |  |

Positions are given considering the 5’ UTR, and then differing from the nomenclature employed in our study in about 185 nucleotides.

References:

8. Moury B, Morel C, Johansen E, Jacquemond M (2002) Evidence for diversifying selection in *Potato virus Y* and in the coat protein of other potyviruses. J Gen Virol 83: 2563-2573.

11. Lorenzen J, Nolte P, Martin D, Pasche JS, Gudmestad NC (2008) NE-11 represents a new strain variant class of *Potato virus Y*. Arch Virol 153: 517-525.

12. Schubert J, Fomitcheva V, Sztangret-Wisniewska J (2007) Differentiation of *Potato virus Y* strains using improved sets of diagnostic PCR-primers. J Virol Methods 140: 66-74.

14. Ogawa T, Tomitaka Y, Nakagawa A, Ohshima K (2008) Genetic structure of a population of *Potato virus Y* inducing potato tuber necrotic ringspot disease in Japan; comparison with North American and European populations. Virus Res 131: 199-212.

15. Mascia T, Finetti-Sialer MM, Cillo F, Gallitelli D (2010) Biological and molecular characterization of a recombinant isolate of *Potato virus Y* associated with a tomato necrotic disease ocurring in Italy. Journal of Plant Pathology 92: 131-138.

17. Hu X, He C, Xiao Y, Xiong X, Nie X (2009) Molecular characterization and detection of recombinant isolates of *Potato virus Y* from China. Arch Virol 154: 1303-1312.

18. Ali MC, Maoka T, Natsuaki T, Natsuaki KT (2010) PVY^NTN-NW^, a novel recombinant strain of *Potato virus Y* predominating in potato fields in Syria. Plant Pathol 59: 31-41.

20. Fanigliulo A, Comes S, Pacella R, Harrach B, Martin DP, et al. (2005) Characterisation of *Potato virus Y* nnp strain inducing veinal necrosis in pepper: a naturally occurring recombinant strain of PVY. Arch Virol 150: 709-720.

25. Visser JC, Bellstedt DU (2009) An assessment of molecular variability and recombination patterns in South African isolates of *Potato virus Y*. Arch Virol 154: 1891-1900.

65. Lorenzen JH, Meacham T, Berger PH, Shiel PJ, Crosslin JM, et al. (2006) Whole genome characterization of *Potato virus Y* isolates collected in the western USA and their comparison to isolates from Europe and Canada. Arch Virol 151: 1055-1074.
