# Supplementary material for: Phylogeography and Molecular Evolution of Potato virus Y
Source: PLoS One. 2012 May 24;7(5):e37853. doi: 10.1371/journal.pone.0037853 (PMC3360008; doi:10.1371/journal.pone.0037853)
Supplement: Table S3 — Genetic diversity estimates per geographic and host structure. (DOC) [file pone.0037853.s006.doc]

|  |  | R1 | | R2 | | R3 | |
| --- | --- | --- | --- | --- | --- | --- | --- |
|  | *n* | *Π* | *SD* | *Π* | *SD* | *Π* | *SD* |
| Global | 59 | 0.1025 | 0.0121 | 0.0854 | 0.0084 | 0.0948 | 0.0060 |
| Japan | 5 | 0.0051 | 0.0010 | 0.0052 | 0.0011 | 0.0051 | 0.0011 |
| North America | 23 | 0.1133 | 0.0174 | 0.0720 | 0.0156 | - | - |
| South Africa | 9 | 0.0350 | 0.0108 | 0.0809 | 0.0239 | 0.0454 | 0.0237 |
| Potato | 54 | 0.0837 | 0.0121 | 0.0752 | 0.0082 | 0.0840 | 0.0056 |
| Tobacco | 2 | 0.0898 | 0.0449 | 0.0555 | 0.0277 | 0.0545 | 0.0272 |

For the three regions analyzed, global nucleotide diversity (*Π*) and standard deviation (*SD*) is given for the full data set and for those significant signatures detected at the population structure analysis. *n* represents the number of isolates.
