# Supplementary material for: Phylogeography and Molecular Evolution of Potato virus Y
Source: PLoS One. 2012 May 24;7(5):e37853. doi: 10.1371/journal.pone.0037853 (PMC3360008; doi:10.1371/journal.pone.0037853)
Supplement: Table S4 — Amino acid composition for potato (P) and non-potato (NP) isolates (55 and five, respectively) at positively selected codons. (DOC) [file pone.0037853.s007.doc]

| **Gene** | **Position** | **P composition** | **NP composition** | **P specific** | **NP specific** |
| --- | --- | --- | --- | --- | --- |
| P1 | 138 | 44H, 8P, 1R, 2S | 2P, 3S | H, R |  |
|  | 247 | 28N, 14H, 10Y, 3S | 4H, 1C | N, Y, S | C |
| P3 | 900 | 34P, 21S | 1P, 3S, 1L |  | L |
|  | 919 | 32N, 12G, 11D | 2N, 2D, 1H | G | H |
|  | 1150 | 35L, 20M | 5L | M |  |
|  | 1404 | 39F, 14H, 2L | 3F, 2Y | H, L | Y |
|  | 1966 | 42T, 11N, 1I, 1 S | 2T, 1N, 2S | I |  |
| NIb | 2508 | 50K, 5R | 2K, 3R |  |  |
| CP | 2922 | 35I, 20V | 4I, 1V |  |  |
|  | 2981 | 33V, 21I, 1N | 1V, 3I, 1H | N | H |
|  | 2987 | 36G, 16V, 3M | 1G, 1M, 2I, 1T | V | I,T |

The last two columns indicate those amino acids that have been detected only in P or NP isolates, respectively, for a given position.
