# Supplementary material for: Phylogeography and Molecular Evolution of Potato virus Y
Source: PLoS One. 2012 May 24;7(5):e37853. doi: 10.1371/journal.pone.0037853 (PMC3360008; doi:10.1371/journal.pone.0037853)
Supplement: Table S5 — Amino acid composition for potato (P) and non-potato (NP) isolates (55 and five, respectively) at covarying codons. (DOC) [file pone.0037853.s008.doc]

| **Gene** | **Position** | **P composition** | **NP composition** | **P specific** | **NP specific** |
| --- | --- | --- | --- | --- | --- |
| P1 | 111 | 45E, 9D, 1S | 4D, 1N | E | N |
|  | 121 | 34R, 11Q, 10P | 5P | R, Q |  |
|  | 214 | 45H, 8R, 1C, 1S | 1R, 3C, 1A | H, S | A |
|  | 241 | 45N, 8T, 2A | 1T, 4A | N |  |
| HC-Pro | 286 | 37S, 18N | 3N, 1D, 1H | S | D, H |
|  | 289 | 46S, 9N | 4N, 1R | S | R |
|  | 305 | 45T, 10S | 4S, 1A | T | A |
|  | 310 | 55V | 1V, 1T, 1L, 1I, 1E |  | T, L, I, E |
|  | 321 | 54V, 1F | 1V, 4I | F | I |
|  | 369 | 54A, 1S | 4S,1M | A | M |
|  | 378 | 35K, 10S, 10N | 3N, 1Y, 1I | K, S | Y, I |
|  | 379 | 54K, 1N | 4K, 1R | N | R |
|  | 395 | 54L, 1K | 3L, 2I | K | I |
|  | 569 | 53N, 1D, 1K | 5N | D, K |  |
|  | 622 | 44R, 10K, 1G | 5K | R, G |  |
|  | 630 | 44I, 10V, 1F | 1I, 4V | F |  |
| P3 | 761 | 40V, 14I, 1E | 4V, 1C | I, E | C |
|  | 901 | 41S, 14H | 4S, 1N | H | N |
|  | 916 | 35N, 17S, 3D | 3D, 2N | S |  |
|  | 919 | 32N, 12G, 11D | 2N, 2D, 1H | G | H |
|  | 936 | 39Y, 16H | 2Y, 2H, 1T |  | T |
|  | 939 | 41R, 14K | 2R, 2K, 1E |  | E |
|  | 943 | 53S, 2Y | 1S, 3Y, 1L |  | L |
|  | 946 | 41R, 14Q | 4R, 1A | Q | A |
|  | 947 | 39Y, 16F | 3Y, 1F, 1S |  | S |
|  | 972 | 38A, 16V, 1T | 3V, 1S, 1I | A, T | S, I |
|  | 974 | 37V, 14R, 2I, 1M 1L | 2R, 2K, 1W | V, I, M, L | K, W |
|  | 983 | 40S, 14N, 1C | 3S, 1N, 1G | C | G |
|  | 1034 | 55A | 3S, 1A, 1T |  | S,T |
|  | 1094 | 54Q, 1H | 4Q, 1K | H | K |
| CI | 1172 | 20T,19I, 15V | 2T, 1I, 2V |  |  |
|  | 1214 | 53H, 2Y | 3H, 1Y, 1Q |  | Q |
|  | 1323 | 38A, 15T, 1P, 1N | 2S, 2N, 1T | A, P | S |
|  | 1404 | 39F, 14H, 2L | 3F, 2Y | H, L | Y |
|  | 1428 | 40K, 14R, 1N | 5K | R, N |  |
|  | 1431 | 41M, 13V, 1I | 5M | V, I |  |
|  | 1631 | 43S, 12G | 3G, 1R, 1S |  | R |
|  | 1639 | 37A, 16V, 2P | 4V, 1A | P |  |
| NIa-Pro | 2058 | 53Y, 1F, 1H | 4F, 1C | Y, H | C |
|  | 2155 | 55I | 3I, 1T, 1V |  | T,V |
| NIb | 2295 | 54T, 1S | 2T, 3A | S | A |
|  | 2373 | 54I, 1V | 4I, 1S | V | S |

The last two columns indicate those amino acids that have been detected only in potato or non-potato isolates, respectively, for a given position.
