# Supplementary material for: Phylogeography and Molecular Evolution of Potato virus Y
Source: PLoS One. 2012 May 24;7(5):e37853. doi: 10.1371/journal.pone.0037853 (PMC3360008; doi:10.1371/journal.pone.0037853)
Supplement: Table S6 — Amino acid composition for PVYN, PVYO and PVYC strain isolates (45, eight and six isolates, respectively) at the subregions showing evidence of positive selection for the branch leading to PVYN isolates respect to the phylogeny of the R1 region. (DOC) [file pone.0037853.s009.doc]

| **Subregion** | **Position** | **PVY^N^**  **composition** | **PVY^O^**  **composition** | **PVY^C^**  **composition** | **PVY^N^**  **specific** | **PVY^O^**  **specific** | **PVY^C^**  **specific** |
| --- | --- | --- | --- | --- | --- | --- | --- |
| 116-124 | 116 | 45L | 8S | 2F, 2I, 2S | L |  | F, I |
|  | 117 | 45E | 8E | 6E |  |  |  |
|  | 118 | 44S, 1L | 6P, 2S | 6P | L |  |  |
|  | 119 | 45Q | 8Q | 6Q |  |  |  |
|  | 120 | 43V, 1A, 1M | 8A | 6T | V, M |  | T |
|  | 121 | 34R, 11Q | 8P | 6P | R, Q |  |  |
|  | 122 | 44R, 1K | 8R | 6K |  |  |  |
|  | 123 | 45G | 8G | 5G, 1R |  |  | R |
|  | 124 | 45V | 7I, 1V | 4V, 2I |  |  |  |
| 160-165 | 160 | 44T, 1I | 8E | 5A, 1D | T, I | E | A, D |
|  | 161 | 45K | 8K | 6K |  |  |  |
|  | 162 | 45G | 8R | 4K, 2R | G |  | K |
|  | 163 | 45G | 8G | 6G |  |  |  |
|  | 164 | 45S | 8S | 6S |  |  |  |
|  | 165 | 45V | 8V | 5V, 1N |  |  | N |
| 205-214 | 205 | 45D | 8D | 5D, 1I |  |  | I |
|  | 206 | 45K | 8M | 4M, 1C, 1T | K |  | C, T |
|  | 207 | 45W | 8W | 5W, 1G |  |  | G |
|  | 208 | 45T | 8T | 5T, 1H |  |  | H |
|  | 209 | 45V | 8V | 3I, 1L, 1S, 1T |  |  | I, L, S, T |
|  | 210 | 35V, 10M | 8G | 4E, 1K, 1S | V, M | G | E, K, S |
|  | 211 | 35R, 10C | 8L | 4C, 1R, 1V |  | L | V |
|  | 212 | 45L | 8L | 5L, 1C |  |  | C |
|  | 213 | 35Q, 10K | 8Q | 5K, 1N |  |  | N |
|  | 214 | 45H | 8R | 3C, 1A, 1R, 1S | H |  | C, A, S |
| 240-245 | 240 | 45S | 8N | 6N | S |  |  |
|  | 241 | 45N | 8T | 1T, 5A | N |  | A |
|  | 242 | 44T, 1A | 8K | 2D, 1A, 1G, 1N, 1W | T | K | D, G, N, W |
|  | 243 | 45N | 8S | 4S, 1K, 1R | N |  | K, R |
|  | 244 | 45L | 8L | 6L |  |  |  |
|  | 245 | 45K | 8K | 3K, 3R |  |  | R |

The last three columns indicate those amino acids that have been detected only in PVY^N^, PVY^O^ or PVY^C^ groups, respectively, for a given position.
